# Prediction and Validation of Immunogenic Domains of Pneumococcal Proteins Recognized by Human CD4^+^ T Cells

**DOI:** 10.1128/IAI.00098-19

**Published:** 2019-05-21

**Authors:** Martijn D. B. van de Garde, Els van Westen, Martien C. M. Poelen, Nynke Y. Rots, Cécile A. C. M. van Els

**Affiliations:** a Centre for Infectious Disease Control, National Institute for Public Health and the Environment, Bilthoven, The Netherlands; Albert Einstein College of Medicine

**Keywords:** CD4^+^ T cells, HLA-DR restriction, MHC, pneumococcus, *Streptococcus pneumoniae*, adaptive immunity, epitope prediction, pneumococcal proteins

## Abstract

CD4^+^ T-cell mechanisms are implied in protection against pneumococcal colonization; however, their target antigens and function are not well defined. In contrast to high-throughput protein arrays for serology, basic antigen tools for CD4^+^ T-cell studies are lacking. Here, we evaluate the potential of a bioinformatics tool for *in silico* prediction of immunogenicity as a method to reveal domains of pneumococcal proteins targeted by human CD4^+^ T cells. For 100 pneumococcal proteins, CD4^+^ T-cell immunogenicity was predicted based on HLA-DRB1 binding motifs. For 20 potentially CD4^+^ T-cell immunogenic proteins, epitope regions were verified by testing synthetic peptides in T-cell assays using peripheral blood mononuclear cells from healthy adults. Peptide pools of 19 out of 20 proteins evoked T-cell responses. The most frequent responses (detectable in ≥20% of donors tested) were found to SP_0117 (PspA), SP_0468 (putative sortase), SP_0546 (BlpZ), SP_1650 (PsaA), SP_1923 (Ply), SP_2048 (conserved hypothetical protein), SP_2216 (PscB), and SPR_0907 (PhtD). Responding donors had diverging recognition patterns and profiles of signature cytokines (gamma interferon [IFN-γ], tumor necrosis factor alpha [TNF-α], interleukin-13 [IL-13], and/or IL-17A) against single-epitope regions. Natural HLA-DR-restricted presentation and recognition of a predicted SP_1923-derived epitope were validated through the isolation of a CD4^+^ T-cell clone producing IFN-γ, TNF-α, and IL-17A in response to the synthetic peptide, whole protein, and heat-inactivated pneumococcus. This proof of principle for a bioinformatics tool to identify pneumococcal protein epitopes targeted by human CD4^+^ T cells provides a peptide-based strategy to study cell-mediated immune mechanisms for the pneumococcal proteome, advancing the development of immunomonitoring assays and targeted vaccine approaches.

## Abstract (precis)

CD4^+^ T-cell mechanisms are implied in protection against pneumococcal colonization; however, their target antigens and function are not well defined. In contrast to high-throughput protein arrays for serology, basic antigen tools for CD4^+^ T-cell studies are lacking.

## INTRODUCTION

Streptococcus pneumoniae (pneumococcus) is a common Gram-positive inhabitant of the human nasopharynx, which is its natural reservoir. There it may reside as a commensal bacterium along with other microorganisms identified on the respiratory epithelium. Such asymptomatic carriage is highest during the first year of life, with rates up to 79%, and progressively declines with age to rates of <10% in adults (1–3). Nasopharyngeal (NP) colonization is a prerequisite for transmission of pneumococci to other individuals and for developing pneumococcal disease. S. pneumoniae is a leading cause of a wide range of infections, including otitis media, community-acquired pneumonia, sepsis, and meningitis (4, 5).

There is widespread evidence that natural colonization is an immunizing event that leads to humoral immunity to capsular polysaccharide (PS) antigens (6). These PS are an important class of virulence factors, of which >90 different serotypes exist (7). PS-specific humoral immunity is highly protective, as is evidenced by the effectiveness of currently licensed pneumococcal conjugate vaccines (PCV) that may contain 10 to 13 different serotypes (8–15). The drawback of PS-based immunity is that it is highly serotype specific and that pneumococci expressing nonvaccine serotype PS can still colonize the PCV-vaccinated host, calling for novel PS-independent vaccines (11–15).

Natural as well as experimental colonization also induces humoral and cell-based immune responses to pneumococcal proteins, a class of more conserved antigens (16–22). Anti-protein antibody responses were shown to protect against invasive pneumococcal disease (23–26), whereas CD4^+^ T-cell-based immunity, in particular that mediated by interleukin-17A (IL-17A)-producing Th17 cells, plays an important role in the prevention of pneumococcal recolonization (18, 25, 27, 28) and experimental pneumonia in mice (29). Recently, Th17 cells mediating responses to pneumococcal protein antigens, being detectable only at low frequencies in peripheral blood mononuclear cells (PBMCs), have also been implied in the protection against colonization in humans (30). Th17 responses are involved in the recruitment and activation of neutrophils, monocytes, and macrophages, which results in quick clearance of opsonized pneumococci by phagocytosis (18, 27, 28).

Unlike antibody responses, CD4^+^ T cells do not recognize whole antigens or conformational epitopes. Instead, they clonally recognize intracellularly degraded fragments of antigens that are presented at the cell surface of antigen-presenting cells (APC) in the peptide-binding groove of self-major histocompatibility complex (MHC) class II molecules. These MHC class II molecules are highly polymorphic. Main human MHC class II molecules implied in CD4^+^ T-cell immunity are human leukocyte antigen (HLA)-DR molecules. These are transmembrane dimers consisting of an alpha and beta chain whose membrane-distal domains together form a peptide-binding groove. Of the functional loci encoding HLA-DR beta chains, the HLA-DRB1 locus is by far the most polymorphic, leading to many HLA-DRB1 alleles in the population that have slightly different binding motifs impacting which peptides become bound and presented to T cells (31–34). The low frequencies of antigen-specific CD4^+^ T cells in PBMCs and this dependence on the presence of MHC class II-matched APC in T-cell assays dictate that large numbers of an individual’s PBMCs are required to screen arrays of pneumococcal proteins for CD4^+^ T-cell recognition. Therefore, as opposed to serology (35–38), the S. pneumoniae antigenome recognized by human CD4^+^ T cells has remained largely unknown, with the exception of a few proteins (18, 39–44). While complicating T-cell studies, MHC binding rules can also help to predict which protein sequences likely become MHC class II molecules presented to T cells, thereby facilitating a selectivity approach in T-cell immunogenicity screening (43, 45, 46).

In the current study, we evaluated proof of principle for a reverse immunology platform to *in silico* predict the T-cell immunogenicity for a semilarge panel of pneumococcal proteins based on HLA-DRB1 binding motifs. Predicted protein regions were validated by *in vitro* assessment of human peripheral T-cell responses to synthetic peptides and whole proteins. We found proof that hitherto unknown specificities and genuine HLA-DR-restricted pneumococcal CD4^+^ T-cell epitopes can be elucidated by bioinformatics. This provides a peptide-based PBMC-saving strategy to study cell-mediated immune mechanisms to S. pneumoniae.

## RESULTS

### Pneumococcal proteins show significant potential for T-cell immunogenicity.

**FIG 1:**
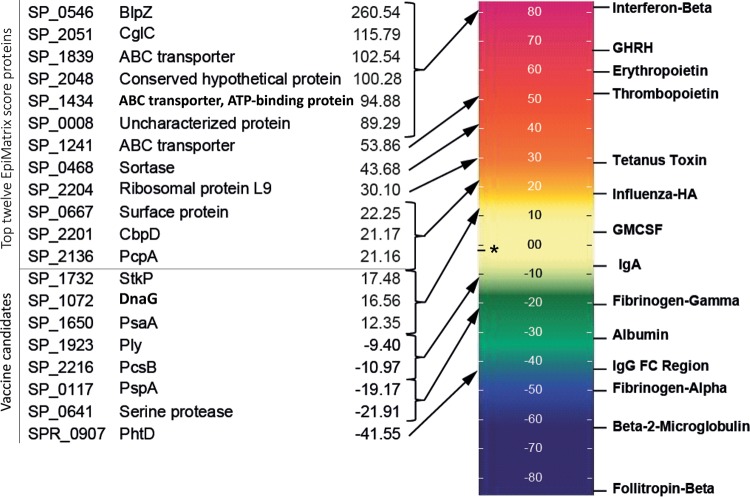
EpiMatrix immunogenicity scale of the 20 selected pneumococcal proteins compared to well-known proteins. The top 12 most immunogenic proteins (with EpiMatrix protein scores of >20) were selected, together with 8 (pre)clinical vaccine candidates with various EpiMatrix protein scores. Their EpiMatrix protein scores are depicted next to the proteins with well-known immunogenicity. An asterisk indicates the average EpiMatrix protein score of all 100 pneumococcal proteins.

A nonsaturating primary list of 100 pneumococcal proteins, likely targeted by the adaptive immune system, was selected from the TIGR4 proteome of >2,000 open reading frames for bioinformatics analysis, mainly based on earlier evidence for B- or T-cell recognition or on their protective potential as a vaccine candidate (Table 1) (35, 36, 41, 43, 44, 47–49). CD4^+^ T cells do not preferentially target surface proteins; therefore, selected proteins of various subcellular localizations, comprising cell wall, cell membrane, cytoplasmic proteins, secreted proteins, and proteins with unknown localization, were included (Table 1). The immunogenic potential of the 100 selected proteins was determined *in silico* based on the binding of potential epitopes to common HLA-DRB1 types and is expressed as an EpiMatrix protein score. Twelve out of 100 proteins showed an EpiMatrix protein score of >20, indicating a significant potential for T-cell immunogenicity. Potential cross-reactivity of frames against the human genome, as determined by a Janus protein score of >3, was limited to a single protein CglC (SP_2051) (Table 1). The 12 proteins with an EpiMatrix protein score above 20, together with eight known (pre)clinical vaccine candidates with various EpiMatrix protein scores, were selected for further analyses (Table 2). Ranking of the selected proteins next to common proteins with known immunogenicity revealed that all vaccine candidate proteins had EpiMatrix protein scores of <20, which was lower than those of, e.g., tetanus toxin and influenza hemagglutinin (Fig. 1). Five out of eight (pre)clinical vaccine candidates even had an EpiMatrix protein score lower than the average (−2.6) for all 100 proteins (Fig. 1). However, such a low score does not exclude the immunogenic potential of dedicated protein regions within a protein. The EpiMatrix system identified 264 putative T-cell immunogenic clusters within the 20 selected proteins. Potential cross-reactivity with human peptides was indicated for 60 clusters with Janus cluster scores above 2.0 (data not shown). Characteristics and sequences of the 2 to 5 potentially most immunogenic regions per protein, selected for peptide synthesis and further *in vitro* immunogenicity analysis, are shown in Table 2.

**TABLE 1:** Characteristics and immunogenicity scores of 100 selected pneumococcal proteins

Gene name	Strain	Protein designation	Length (amino acids)	Protein name	Localization^*a*^	No. of EpiMatrix hits	EpiMatrix protein score	Janus protein score	Reference
SP_0546	TIGR4	BlpZ	77	BlpZ protein, fusion	Cell membrane	96	260.54	0.75	48
SP_2051	TIGR4	CglC	108	Competence protein	Unknown	86	115.79	4.78	36
SP_1839	TIGR4		583	Putative ABC transporter ATP-binding protein exp8	Cell membrane	480	102.54	2.39	44
SP_2048	TIGR4		153	Conserved hypothetical protein	Cell membrane	113	100.28	2.42	36
SP_1434	TIGR4		586	ABC transporter, ATP-binding protein	Mitochondral membrane	456	94.88	1.51	44
SP_0008	TIGR4		122	Uncharacterized protein	Cell membrane	89	89.29	1.25	36
SP_1241	TIGR4		721	Amino acid ABC transporter, amino acid-binding protein	Cell membrane	466	53.86	1.33	35, 36
SP_0468	TIGR4		283	Putative sortase	Cell membrane	165	43.68	2.45	36
SP_2204	TIGR4	RplI	150	Ribosomal protein L9	Ribosome	78	30.1	1.49	36
SP_0667	TIGR4		328	Pneumococcal surface protein, putative	Cell membrane	166	22.25	0.56	35, 36
SP_2201	TIGR4	CbpD	448	Choline-binding protein D	Cell wall	222	21.17	0.52	35, 36
SP_2136	TIGR4	PcpA	621	Choline-binding protein A	Cell wall	319	21.16	1.03	35, 36, 44, 47
SP_0348	TIGR4	CpsC	230	Capsular polysaccharide biosynthesis protein	Cell membrane	114	18.68	2.68	36
SP_1759	TIGR4	SecA-2	790	Preprotein translocase, SecA subunit	Cell membrane, cytoplasm	395	18.25	1.09	36
SP_0770	TIGR4		513	ABC transporter, ATP-binding protein	Unknown	254	18.14	1.98	36
SP_1732	TIGR4	StkP	659	Serine/threonine protein kinase	Cell membrane	331	17.48	2.34	35, 36, 40
SP_1072	TIGR4	DnaG	586	DNA primase	Primosome	284	16.55	1.13	44
SP_0466	TIGR4		279	Sortase, putative	Cell membrane	133	16.05	1.54	36
SP_2239	TIGR4	HtrA	393	Serine protease	Unknown	191	15.27	1.08	35, 36
SP_0529	TIGR4	BlpC	453	BlpC ABC transporter	Mitochondral membrane	222	14.24	1.52	35, 36
SP_0369	TIGR4	PonA	719	Penicillin-binding protein 1A	Secreted	346	14.19	1.8	36
SP_0378	TIGR4	CbpJ	328	Choline-binding protein J	Cell wall	158	13.86	0.24	36
SP_1650	TIGR4	PsaA	309	Manganese ABC transporter substrate-binding lipoprotein	Cell membrane	145	12.35	1.46	35, 36
SP_0222	TIGR4	RpsN	89	Ribosomal protein S14	Ribosome	40	11.6	0.88	36
SP_1676	TIGR4		305	*N*-Acetylneuraminate lyase, putative	Unknown	147	11.3	0.78	36
SP_1954	TIGR4		467	Serine protease, subtilase family	Unknown	214	11.09	0.98	36
SP_0197	TIGR4		416	Dihydrofolate synthetase, putative	Unknown	200	10.34	1.62	36
SP_2128	TIGR4		285	Transketolase, N-terminal subunit	Unknown	136	9.64	1.01	36
SP_2021	TIGR4		469	Glycosyl hydrolase	Unknown	213	7.58	0.97	36
SP_0377	TIGR4	CbpC	340	Choline-binding protein C	Cell wall	155	7.38	0.39	35, 36
SP_1980	TIGR4	Cbf1	308	Cmp-binding factor 1	Unknown	140	6.07	1.9	36
SP_0609	TIGR4		254	Amino acid ABC transporter, amino acid-binding protein	Unknown	111	4.71	1.04	36
SP_0613	TIGR4	RnJ	553	Ribonuclease J	Cytoplasm	244	4.22	1.31	36
SP_1891	TIGR4	AmiA	659	Oligopeptide ABC transporter	Cell membrane	296	3.8	1.34	35, 36
SP_2099	TIGR4	Pbp1B	821	Penicillin-binding protein 1B	Cell membrane	361	2.41	1.79	36
SP_1527	TIGR4	AliB	652	Oligopeptide ABC transporter	Cell membrane	289	1.56	1.46	35, 36
SP_0390	TIGR4	CbpG	285	Choline-binding protein G	Cell wall	119	1.52	0.71	36
SP_0688	TIGR4	MurD	450	UDP-*N*-acetylmuramoylalanine–d-glutamate ligase	Cytoplasm	193	−0.52	1.12	36
SP_2194	TIGR4		810	ATP-dependent Clp protease, ATP-binding subunit	Unknown	348	−0.83	1.19	36
SP_1687	TIGR4	NanB	697	Neuraminidase B	Unknown	295	−1.17	1	35, 36
SP_1553	TIGR4		623	ABC transporter, ATP-binding protein	Cytoplasm	270	−1.42	2.11	44
SP_1573	TIGR4	LytC	490	Lysozyme	Cell wall	206	−2.59	0.7	35, 36
SP_2039	TIGR4		207	Conserved hypothetical protein	Unknown	85	−4.93	0.89	36
SP_2141	TIGR4		626	Glycosyl hydrolase-related protein	Unknown	255	−5.03	1.05	36
SP_1227	TIGR4		234	DNA-binding response regulator	Unknown	94	−5.9	1.63	36
SP_0509	TIGR4	HsdM	487	Type I restriction-modification system, M subunit	Unknown	198	−6.52	1.55	36
SP_0251	TIGR4		812	Formate acetyltransferase, putative	Cytoplasm	331	−7.39	1.34	36
SP_1221	TIGR4		1,084	Type II restriction endonuclease	Unknown	438	−7.58	1.57	36
SP_1124	TIGR4	GlgA	477	Glycogen synthase	Unknown	197	−7.99	0.65	36
SP_0330	TIGR4	RegR	333	Sugar-binding transcriptional regulator	Unknown	133	−8.8	0.92	36
SP_1999	TIGR4	CcpA	336	Catabolite control protein A	Unknown	133	−9.07	1.08	36
SP_1923	TIGR4	Ply	471	Pneumolysin	Secreted, cell membrane	188	−9.4	0.8	36, 47, 49
SP_0981	TIGR4	PrsA	313	Foldase protein	Cell membrane	122	−9.91	2.07	35, 36
SP_0295	TIGR4	RpsI	130	Ribosomal protein S9	Ribosome	49	−9.95	1.02	36
SP_2216	TIGR4	PcsB	392	Secreted 45-kDa protein Usp45	Secreted	152	−10.97	2.43	35, 36, 44
SP_0071	TIGR4	ZmpC	1,856	Immunoglobulin A1 protease, zinc metallose C	Secreted, cell wall	721	−12.03	1.25	35, 36
SP_1032	TIGR4	PiaA	341	Iron compound ABC transporter	Periplasm	128	−12.47	1.16	35, 36
SP_0148	TIGR4		276	ABC transporter, substrate-binding protein	Unknown	102	−12.68	1.12	36
SP_1283	TIGR4		107		Cell membrane	40	−13.02	0.9	36
SP_0212	TIGR4	RplB	277	Ribosomal protein L2	Ribosome	106	−13.12	1.28	36
SP_0749	TIGR4	LivJ	386	Branched-chain amino acid ABC transporter	Periplasm	148	−13.59	1.28	35, 36
SP_0785	TIGR4		399	Conserved hypothetical protein	Cell membrane	152	−17.42	1.36	35, 36
SP_1154	TIGR4	ZmpA	2,004	IgA1 protease	Secreted, cell wall	739	−18.31	1.4	35, 36
SP_0664	TIGR4	ZmpB	1,906	Zinc metallose B (putative)	Secreted, cell wall	684	−18.87	1.02	35, 36
SP_0930	TIGR4	CbpE	627	Choline binding protein E	Cell wall	223	−18.91	1.41	35, 36
SP_0117	TIGR4	PspA	744	Pneumococcal surface protein A	Cell wall	272	−19.17	0.95	35, 36, 43
SP_1287	TIGR4	Ffh	523	Signal recognition particle protein	Cytoplasm	187	−19.59	1.15	36
SP_1175	TIGR4		802	Conserved domain protein	Unknown	282	−20.34	1.4	35, 36
SP_0057	TIGR4	StrH	1,312	Beta-*N*-acetylhexosaminidase	Secreted, cell wall	473	−20.55	0.96	35, 36
SP_0943	TIGR4	Gid	444	Gid protein	Cytoplasm	162	−20.85	0.52	36
SP_1330	TIGR4	NanE	233	*N*-Acetylmannosamine-6-P epimerase, putative	Unknown	80	−21.28	1.28	36
SP_1804	TIGR4		202	General stress protein 24, putative	Unknown	67	−21.42	1.64	36
SP_1522	TIGR4		205	Conserved domain protein	Unknown	70	−21.87	1.23	36
SP_0641	TIGR4		2,140	Serine protease	Cell membrane, cell wall	744	−21.91	0.88	35, 36, 44
SPR0561	R6	PrtA	2,144	Cell wall-associated proteinase	Cell membrane, cell wall	727	−24.14	0.84	35, 36
SP_1888	TIGR4	AmiE	355	Oligopeptide ABC transporter, ATP-binding protein	Cell membrane	116	−26.07	1.74	36
SP_2092	TIGR4	GalU	299	UTP-glucose-1-phosphate uridylyltransferase	Unknown	98	−26.17	0.77	36
SP_0463	TIGR4		665	Cell wall surface anchor family protein	Cell membrane, cell wall, secreted	217	−26.65	1.82	36
SP_1661	TIGR4	DivIVA	262	Cell division protein	Cytoplasm	87	−27.6	0.97	36
SP_2190	TIGR4	CbpA	693	PspC/choline-binding protein A	Secreted	227	−28.02	0.45	35, 36
SP_0368	TIGR4	GH101	1,767	Cell wall surface anchor family protein	Secreted, cell wall	589	−28.22	1.14	35, 36
SP_1735	TIGR4	Fmt	311	Methionyl-tRNA formyltransferase	Unknown	99	−28.56	0.61	36
SP_1991	TIGR4		257	Putative hydrolase	Unknown	81	−29.27	1.07	36
SP_0082	TIGR4		857	Cell wall surface anchor protein	Secreted, cell wall	273	−31.05	1.25	35, 36
SP_0069	TIGR4	CbpI	211	Choline-binding protein I	Cell wall	61	−35.15	0.25	36
SP_0239	TIGR4		445	Conserved hypothetical protein	Unknown	130	−37.2	1.3	36
SP_0498	TIGR4		1,659	Endo-β-*N*-acetylglucosaminidase, putative	Secreted, cell wall	486	−38.88	1.3	35, 36
SP_0648	TIGR4	BgaA	2,233	β-Galactosidase	Secreted, cell wall	633	−39.14	0.98	35, 36
SP_0519	TIGR4	DnaJ	378	DnaJ protein	Cytoplasm	107	−39.26	1.85	36
SP_1174	TIGR4		819	Conserved domain protein	Unknown	228	−39.36	1.41	35, 36
SP_1429	TIGR4		428	Peptidase, U28 family	Unknown	123	−39.75	0.72	36
SPR0907	R6	PhtD	853	Pneumococcal histidine triad protein D	Cell membrane	235	−41.55	1.59	35, 36, 47
SP_1478	TIGR4		280	Oxidoreductase, aldo/ketoreductase family	Unknown	74	−42.22	1.11	36
SP_2108	TIGR4	MalX	423	Maltose ABC transporter	Cell membrane	113	−43.4	1.07	35, 36
SP_1664	TIGR4	SepF	179	Cell division protein	Cytoplasm	47	−43.76	0.4	36
SP_0107	TIGR4	LysM	195	Domain protein	Unknown	46	−50.7	0.93	35, 36
SP_1937	TIGR4	LytA	318	Autolysin	Secreted	69	−54.36	0.9	35, 36
SP_1374	TIGR4	AroC	388	Chorismate synthetase	Unknown	85	−56.28	0.64	36
SP_1992	TIGR4		221	Cell wall surface anchor family protein	Cell membrane	41	−64.4	0.95	36
SP_1772	TIGR4		4,776	Cell wall surface anchor family protein	Cell membrane	303	−96.41	1.11	36

a Subcellular localization based on UniprotKB database.

**TABLE 2:** Characteristics of immunogenic regions in the selected proteins and vaccine candidates

Ranking in EpiMatrix protein score^*a*^	Gene name	Protein name/designation	Cluster address^*b*^	Cluster sequence^*c*^	Synthesized peptides (location)	Hydrophobicity^*d*^	EpiMatrix hits^*e*^^,^^*f*^	EpiMatrix cluster score^*f*^^,^^*g*^	Janus cluster score
1	SP_0546	BlpZ	30–57	FNV**FVLTFVSAVVFNFLNSMLALMA**IFI	30–47, 40–57	2.09	39	76.27	0.34
			58–77	GAG**YVVGFWLLILNENQRA**N	60–77	0.27	20	36.19	2.25
			10–32	SKT**LDRLTPYILVLASDTIA**FNV	10–27, 15–32	0.59	14	21.17	0.31
			1–18	M**YKHLFFLDSKTLDR**LTP	1–18	−0.27	11	18.34	0.55
			46–60	LNS**MLALMAIFI**GAG	46–60	1.82	6	11.24	0.27
2	SP_2051	CglC	20–46	EM**LVVLLIISVLFLLFVPNLTKQK**EAV	20–37, 29–46	1.52	43	80.29	7.64
			1–24	**MKKMMTFLKKAKVKAFTLVEM**LVV	1–18, 7–24	0.7	22	36.63	2.22
3	SP_1839	Putative ABC transporter ATP-binding protein exp8	164–194	LTA**LVLLFLPLIFLLVNLYRKKSVKIIE**KTR	164–181, 172–189, 177–194	1	56	106.56	5.06
			11–43	LKR**LMSYLKPYGLLTFLALSFLLATTVIKS**VIP	6–23, 19–36, 25–42	1.08	43	71.09	5.8
			74–97	LQT**VVQYVGNLLFARVSYSIV**RDI	74–91, 80–97	0.74	29	48	1.28
			136–162	FSG**ILSSFISAVFIFLTTLYTMLV**LDF	136–153, 154–162	1.78	26	44.08	0.97
			243–267	ALD**ALFLRPAMSLLKLLGYAVL**MAY	243–260, 250–267	1.32	25	40.49	1.3
4	SP_2048	Conserved hypothetical protein	23–47	LLA**LIVISGGLLLFQAMSQLLI**SEV	23–40, 30–47	1.88	27	53.31	3.38
			8–34	QSK**SHKVKAFTLLESLLALIVISG**GLL	8–25, 17–34	0.88	27	52.34	4.56
			129–145	LVR**FHFQFQKGLER**EFI	129–145	−0.18	11	21.26	0.09
			108–127	GRG**YQPMVYGLKSVRIT**EDN	109–126	−0.76	12	18.91	0.42
			98–112	SDD**FRKTNARGR**GYQ	98–112	−2.03	7	12.73	0.14
5	SP_1434	ABC transporter, ATP-binding protein	259–288	IIP**IVYFMTSLASAKVILLELIMILFL**SGV	260–277, 271–288	2.05	36	59.16	0.93
			15–40	DKK**YLGVLAIIFSAISAALTVYG**YYL	15–32, 23–40	1.06	32	55.21	2.91
			35–61	VYG**YYLIYKFLDKLIINSNLSGAE**SIA	35–52, 44–61	0.5	28	50.67	2.68
			151–174	ALG**FIVSIRVGIILLALTIIG**GLI	151–168, 157–174	2.4	23	39.93	4.34
			513–538	QKA**FKNLMKDKTVIMIAHRLSTI**KDL	513–530, 521–538	−0.16	21	34.1	0.96
6	SP_0008	Uncharacterized protein	30–57	RNR**FMGGVLILIMLLFILPTFNLAQ**SYQ	33–50, 40–57	0.94	29	45.06	2.13
			2–17	SKN**IVQLNNSFIQ**NEY	2–17	−0.72	16	33.86	0.56
			47–68	LPT**FNLAQSYQQLLQRRQQ**LAD	47–64, 51–68	−0.73	17	32.05	1.28
			96–116	AAK**YTRAKYYYSKSREKV**YTI	97–114	−1.12	11	15.5	0.45
7	SP_1241	Amino acid ABC transporter, amino acid-binding protein	694–720	MYA**ILAIFYLVIITLLTRLAKRLE**KRI	694–711, 703–720	1.14	36	66.02	3.45
			508–531	QNN**YKQLLSGLGITLALALIS**FAI	508–525, 514–531	0.93	23	47.43	3.12
			1–23	**MKKKFLAFLLILFPIFSLGI**AKA	1–18, 6–23	1.42	26	44.64	3.52
			532–558	AIV**IGIIFGMFSVSPYKSLRVISE**IFV	533–550, 541–558	1.51	24	36.64	0.88
			277–293	FAP**FVFQNSSNQYT**GID	277–293	−0.25	15	32.08	1
8	SP_0468	Putative sortase	259–283	RGL**VVLAFLGILFVLWKLARLLRG**K	259–276, 266–283	1.33	38	67.95	4.29
			3–26	RTK**LRALLGYLLMLVACLIPI**YCF	3–20, 9–26	1.45	23	44.98	5.1
			91–114	PDA**VYGYLSIPSLEIMEPVYL**GAD	92–109	0.38	13	19.93	0.77
			49–73	TEM**YQEQQNHSLAYNQRLASQN**RIV	49–66, 56–73	−1.21	12	16.48	0.71
			40–55	HAT**FVKSMTTEMY**QEQ	40–55	−0.79	9	16.34	0
9	SP_2204	RplI	22–42	PTG**YAQNFLIKKNLAKEA**TAQ	23–40	−0.56	18	31.83	3.11
			130–150	DVP**VKIYQDITSVINLRVKEG**	133–150	−0.02	17	27.31	1.24
			101–121	AEE**LQKQFGIKIDKRHIQ**VQA	102–119	−0.78	12	19.1	0.25
			1–20	MK**VIFLADVKGKGKKGE**IKE	1–18	−0.37	8	11.6	2.13
			89–103	GRT**FGSITNKKI**AEE	89–103	−0.81	6	11.45	1.43
10	SP_0667	Pneumococcal surface protein, putative	293–319	AKS**YNSLFHMSKKRMYRQLTSDFD**KFS	293–310, 302–319	−1.02	20	33	0.71
			154–174	KNA**WQGAYYLKSNGKMAQ**GEW	156–173	−1.17	16	28.72	0.38
			73–97	KGA**FKAKQSTAIQINTSSATTS**GWV	73–90, 80–97	−0.27	15	22.93	0.2
			1–19	M**NKRLFSKMSLVTLPI**LAL	1–18, 2–19	0.86	15	22	0.44
			265–282	DGV**WKEVQASTASSS**NDS	265–282	−0.86	12	21.6	1.58
11	SP_2201	CbpD	10–30	GTS**YYLKMSVKKLVPFLV**VGL	11–28	0.84	21	42.72	1.64
			351–375	TVG**WKKINGSWYHFKSNGSKST**GWL	351–368, 358–375	−0.83	16	29.66	0.56
			237–260	YTA**YNGSYRYVQLEAVNKNPL**GNS	237–254, 243–260	−0.82	18	27.21	0.24
			75–94	CTS**FVAFRLSNVNGFEI**PAA	75–92	0.69	12	21.91	0.92
			313–334	YTA**YNGSRRYIQLEGVTSS**QNY	313–330, 317–334	−1	12	19.78	0.79
12	SP_2136	PcpA	180–205	TSA**FSFSQKLKKLTFSSSSKLEL**ISH	180–197, 188–205	−0.14	27	47.43	2.11
			220–242	PKS**VKTLGSNLFRLTTSLKH**VDV	220–237, 225–242	−0.1	19	35.03	2.19
			285–307	LAS**YSFNKNSYLKKLELNEG**LEK	285–302, 290–307	−0.8	19	30.72	1.7
			205–228	HEA**FANLSNLEKLTLPKSVKT**LGS	205–223, 211–228	−0.21	18	27.09	1.14
			420–440	SEH**IKDVLKSNLSTSNDI**IVE	422–439	−0.3	15	23.65	2.8
16	SP_1732	StkP^*h*^	342–365	KMR**YLILLASLVLVAASLIWI**LSR	342–359, 348–365	1.6	39	80.67	7.24
			262–288	VSE**MYVDLSSSLSYNRRNESKLIF**DET	262–279, 271–288	−0.61	24	37.72	1.25
			111–135	EEA**VRIMGQILLAMRLAHTRGI**VHR	111–128, 118–135	0.24	22	36.66	1.82
			243–266	LEN**VIIKATAKKLTNRYRSVS**EMY	243–260, 249–266	−0.41	16	24.29	1.44
			219–236	TIA**LQHFQKPLPSVI**AEN	219–236	0.08	13	22.85	3.54
17	SP_1072	DnaG^*h*^	381–403	QIE**FLEKIAPLIVQEKSIAA**QNS	381–398, 386–403	0.11	19	31.04	0.85
			16–37	IVE**VIGDVISLQKAGRNYL**GLC	16–33, 20–37	0.73	16	26.88	1.18
			446–468	TMP**VTKQLSAIMRAEAHLLY**RMM	446–463, 451–468	0.21	15	25.87	2.73
			289–309	REH**VEHLKRLTKKLVLVY**DGD	289–306, 292–309	−0.75	14	24.52	2.64
			569–586	DTA**LEELERLISQKRRME**	569–586	−1.13	14	23.52	1.71
23	SP_1650	PsaA^*h*^	1–22	**MKKLGTLLVLFLSAIILVA**CAS	1–18, 5–22	1.92	36	67.66	3.53
			138–161	PHA**WLNLENGIIFAKNIAKQL**SAK	138–155, 144–161	−0.05	21	35.46	0.38
			30–52	GQK**LKVVATNSIIADITKNI**AGD	30–47, 35–52	0.15	13	19.05	1.38
			205–222	EGA**FKYFSKAYGVPS**AYI	205–222	0.03	10	18.83	0.5
			289–309	GDS**YYSMMKYNLDKIAEG**LAK	289–306, 292–309	−0.6	11	17.32	0.09
52	SP_1923	Ply^*h*^	40–61	PDE**FVVIERKKRSLSTNTS**DIS	40–57, 44–61	−0.75	21	35.85	1.86
			405–425	TAH**FTTSIPLKGNVRNLS**VKI	405–422, 408–425	0.07	14	25.56	1.35
			231–253	ERP**LVYISSVAYGRQVYLKL**ETT	231–248, 236–253	−0.09	17	25.45	0.29
			244–259	RQV**YLKLETTSKS**DEV	244–259	−0.91	9	19.2	0.89
			6–28	VND**FILAMNYDKKKLLTHQG**ESI	6–23, 11–28	−0.35	12	17.19	0.5
			173–191	GNS**LDIDFNSVHSGEK**QIQ	174–191	−0.79	8	10.98	0.44
55	SP_2216	PcsB^*h*^	1–20	**MKKKILASLLLSTVMVS**QVA	1–18	1.07	25	43.59	8.04
			114–135	NGA**VTSYINTIVNSKSITE**AIS	117–134	0.23	15	24.26	0.47
			140–161	MSE**IVSANNKMLEQQKADK**KAI	140–157, 144–161	−0.7	13	22.53	0.62
			55–76	VDQ**IQEQVSAIQAEQSNLQ**AEN	55–72, 59–76	−0.77	12	21.29	1.58
			246–268	QQS**VLASANTNLTAQVQAVS**ESA	246–263, 251–268	0	13	18.55	0.23
66	SP_0117	PspA^*h*^	673–690	NGS**WYYLNANGSMAT**GWV	673–690	−0.27	21	41.33	0.1
			1–19	MNK**KKMILTSLASVAI**LGA	2–19	0.84	18	31.98	2.11
			593–610	SDK**WYYVNSNGAMAT**GWL	593–610	−0.47	13	23.92	0
			225–242	QHQ**VDNLKKLLAGAD**PDD	225–242	−1.02	9	15.59	1.44
			185–204	KYD**YATLKVALAKKEVE**AKE	185–204	−0.68	8	10.91	1.13
74	SP_0641	Serine protease^*h*^	378–399	GEK**YWQAIRALRKAGIPMV**VAT	378–395, 382–399	0.05	18	35.22	1.33
			1085–1103	REH**FIRGILNSKSNDA**KGI	1085–1102	−0.76	17	33.69	0.44
			204–221	EEA**IDYLKSINAPFG**KNF	204–221	−0.41	16	32.28	0.38
			138–158	EKA**IKELSSLKNTKVLYT**YDR	139–156	−0.85	16	29.18	3.06
			913–936	MEA**LNSNGKKINFQPSLSMPL**MGF	913–930, 919–936	−0.11	17	28.98	0.38
92	SPR_0907	PhtD^*h*^	805–830	DSS**IRQNAVETLTGLKSSLLLGT**KDN	805–822, 813–830	−0.38	18	28.39	2.89
			606–624	AEA**IYNRVKAAKKVPL**DRM	607–624	−0.44	14	27.87	0.86
			1–22	**MKINKKYLAGSVAVLALSV**CSY	1–18, 5–22	0.8	15	22.03	4.13
			834–853	SAE**VDSLLALLKESQPTPI**Q	835–852	−0.01	13	20.54	3.46
			618–641	KVP**LDRMPYNLQYTVEVKNGS**LII	618–635, 624–641	−0.11	11	14.42	0.25

a Ranking number within the list of 100 pneumococcal proteins in Table 1 based on EpiMatrix protein score.

b The cluster address is the location of the peptide within the protein sequence; clusters are ranked according to their EpiMatrix cluster score.

c The identified core peptides (in boldface) are depicted within N- and C-terminal flanks (not in boldface), which are required for further analysis in immunoassays.

d Hydrophobicity scores of 2 and above are predictive of difficulty synthesizing peptides.

e EpiMatrix hits is the number of Z scores above 1.64.

f EpiMatrix cluster score derives from the number of hits normalized for the length of the cluster and thus is the excess or shortfall in predicted aggregate immunogenicity to a random peptide standard.

g Without flanks.

h Preclinical or clinical vaccine candidate.

### Predicted immunogenic HLA class II peptides induce T-cell proliferation in healthy donors.

**FIG 2:**
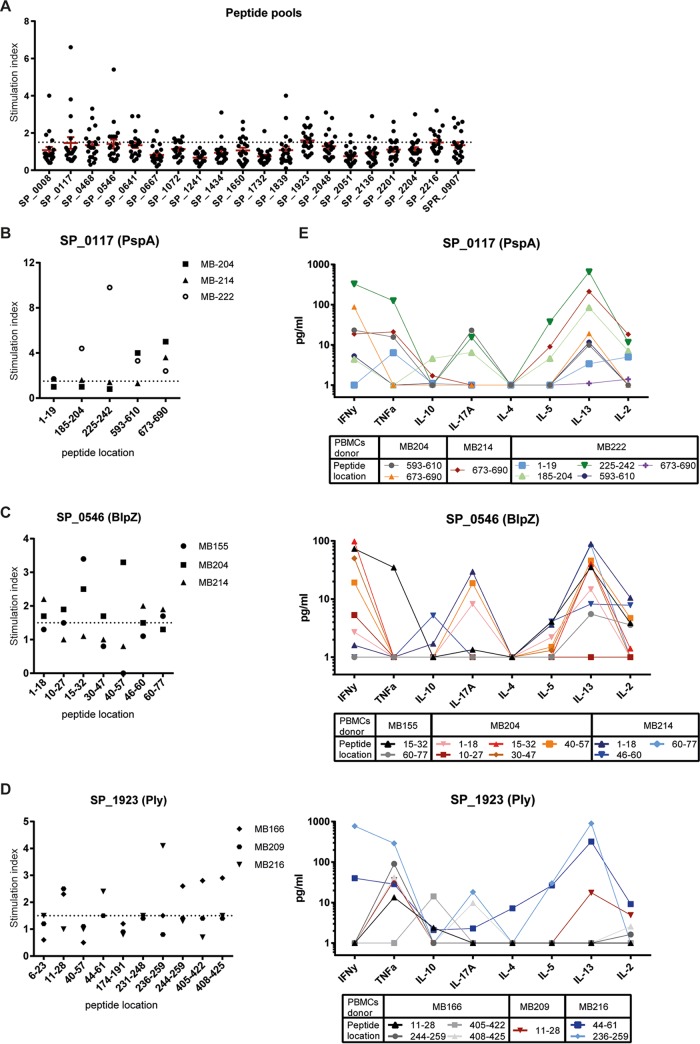
Immunogenicity screening of peptides using PBMCs from healthy adult donors. (A) Proliferation of healthy donor PBMCs after *in vitro* stimulation with peptide pools comprising the most immunogenic regions of the 20 selected proteins was measured. (B to D) In-depth analysis of potential immunogenic individual peptides of the protein PspA (B), BlpZ (C), or Ply (D) assessed in three donors responsive to respective peptide pools. Levels of cytokines produced present in the supernatants of donor PBMCs after single-peptide stimulation are illustrated using a unique colored line/symbol combination per donor/peptide combination, as indicated. (E) Different cytokine responses per donor/peptide stimulation are depicted with interconnecting lines for rapid visual evaluation but have no biological meaning. *y* axes indicate the stimulation index (fold proliferation over medium background) (A to D), and *x* axes depict tested peptide pools (A) or individual peptide locations within the protein (B to D), as indicated.

PBMCs of 21 healthy donors expressing at least one of the HLA-DRB1 alleles in the EpiMatrix system were stimulated with pools of synthetic peptides representing the selected most immunogenic regions per protein (Table 2). The amino acid ABC transporter (SP_1241) protein peptide pool was not able to induce proliferation in any of the 21 donors tested. All other protein peptide pools induced proliferation in up to 10 of the 21 donors (Fig. 2A and Table 3). The highest stimulation indices (SIs) were found for PspA (SP_0117), BlpZ (SP_0546), putative ABC transporter ATP-binding protein exp8 (SP_1839), an uncharacterized protein (SP_0008), and a putative sortase (SP_ 0468). Pneumolysin (SP_1923) showed the highest proliferative responses on average, coinciding with the highest percentage of responders (Fig. 2A and Table 3).

**TABLE 3:** Overview of responders to peptide pools derived from the 20 selected pneumococcal proteins

Gene name	Protein name	No. of responders^*a*^ after peptide pool stimulation (*n* = 21)	% of responders
SP_0008	Uncharacterized protein	3/21	14.3
SP_0117	PspA	5/21	23.8
SP_0468	Putative sortase	6/21	28.6
SP_0546	BlpZ	7/21	33.3
SP_0641	Serine protease	4/21	19.0
SP_0667	Pneumococcal surface protein, putative	1/21	4.8
SP_1072	DnaG	2/21	9.5
SP_1241	Amino acid ABC transporter, amino acid-binding protein	0/21	0.0
SP_1434	ABC transporter, ATP-binding protein	2/21	9.5
SP_1650	PsaA	5/21	23.8
SP_1732	StkP	1/21	4.8
SP_1839	Putative ABC transporter, ATP-binding protein Exp8	3/21	14.3
SP_1923	Ply	10/21	47.6
SP_2048	Conserved hypothetical protein	5/21	23.8
SP_2051	CglC	1/21	4.8
SP_2136	PcpA	3/21	14.3
SP_2201	CbpD	2/21	9.5
SP_2204	RplI	2/21	9.5
SP_2216	PcsB	7/21	33.3
SPR_0907	PhtD	5/21	23.8

a Stimulation index, ≥1.7.

### Diverse T helper cell responses induced by individual immunogenic peptides.

The immunogenicity per whole protein or peptide pool does not discriminate between the potential of single immunodominant epitopes. Therefore, responses at the single epitope level were investigated for PspA, BlpZ, and Ply using PBMCs from 3 out of the top 5 responders to corresponding peptide pools. Proliferation and cytokine secretion were measured after stimulation with individual peptides. Single peptides from PspA induced proliferation in one or more donors tested. PspA_225–242_ showed very strong stimulation of PBMCs of donor MB222 but did not show a response in the other two. In contrast, PspA_673–690_ induced proliferation in all three donors (Fig. 2B). The overlapping BlpZ_30–47_ and BlpZ_40–57_ peptides showed proliferation in a single donor, with the more dominant response being to BlpZ_40–57_. The other peptides of BlpZ induced proliferation in 2/3 donors (Fig. 2C). Two out of 10 single Ply peptides did not activate the PBMCs of any donor. The largely overlapping peptides Ply_405–422_ and Ply_408–425_ activated PBMCs of only a single donor. The strongest proliferative response was found after stimulation with Ply_236–259_ (Fig. 2D).

T-cell activation after stimulation with single PspA, BlpZ, and Ply peptides was also evident by the detection of Th1 (gamma interferon [IFN-γ] and tumor necrosis factor alpha [TNF-α]), Th17 (IL-17A), and/or Th2 (IL-5 and IL-13) T helper type signature cytokines after 5 days in the culture supernatants from the tested donors (Fig. 2E). No or limited amounts of IL-4 and IL-10 were detected. IFN-γ was found in responses to most BlpZ peptides, and only a single peptide induced TNF-α secretion. Conversely, all but one Ply peptide induced TNF-α, with only two peptides inducing IFN-γ. Th2 responses were most abundant against PspA and BlpZ peptides. Th17 responses were detected in response to 3/8 PspA, 4/10 BlpZ, and 3/7 Ply peptide stimulations (Fig. 2E). Notably, single-peptide specificities could evoke the production of one or a combination of (two or three) of the Th1, Th2, and Th17 signature cytokines. For peptides tested in multiple donors, these patterns could be comparable (e.g., for PspA_673–690_ in donors MB204 and MB214) or dissimilar (e.g., for Ply_11–28_ in donors MB166 and MB209).

### Isolation of a CD4^+^ T-cell clone to a predicted Ply epitope.

**FIG 3:**
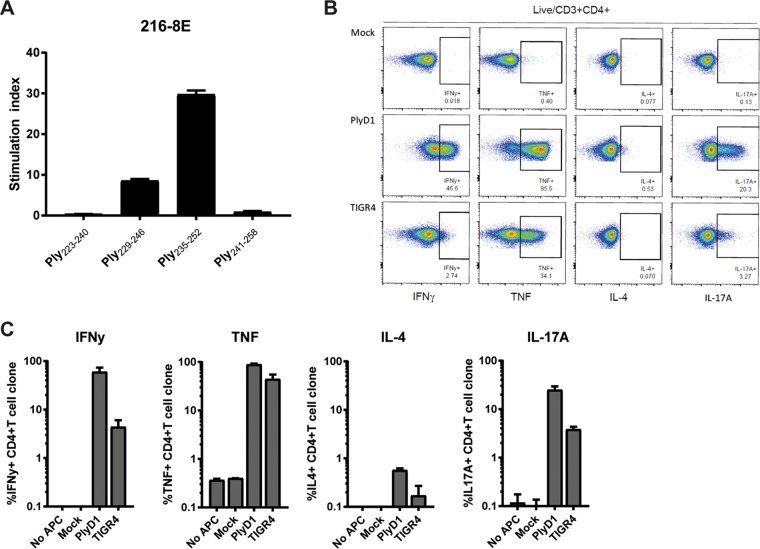
Th1/Th17 dominated responses of CD4^+^ T-cell clone 216-8E to a predicted pneumolysin epitope. (A) The specificity of the isolated CD4^+^ T-cell clone 216-8E for Ply_235–252_ was assessed by measuring T-cell proliferation after stimulation with synthetic 12-mer overlapping Ply peptides. (B) Representative FACS plots of intracellular flow cytometric analysis of singlet live/CD3^+^/CD4^+^ 216-8E cells 6 h after exposure to autologous moDCs pulsed with whole pneumolysoid protein, heat-inactivated TIGR4_ΔCPS_, or medium (Mock). (C) Percentages of 216-8E cells stained positive for IFN-γ, TNF-α, IL-4, or IL-17A, using pooled data from three independent experiments.

To study if the reverse immunology strategy can identify truly processed and presented immunodominant CD4^+^ T-cell epitopes, we stimulated PBMCs from an HLA-DRB1*15- and HLA-DRB1*04-typed donor responding to the Ply peptide pool with a detoxified whole Ply and cloned the responding bulk culture by limiting dilution. We isolated a CD4^+^ T-cell clone (named 216-8E) showing strong proliferative capacity in response to a predicted Ply_235–252_ epitope and a weak or no response to neighboring peptides Ply_229–246_ and Ply_241–258_ (Fig. 3A), providing proof of principle for the reverse immunology approach. The T-cell clone 216-8E recognizes the Ply_235–252_ epitope in the context of HLA-DR, as illustrated by reduced proliferation by adding anti-HLA-DR but not anti-HLA-DQ or anti-HLA-DP antibody (see Fig. S2A in the supplemental material). More specifically, as found after four-digit HLA typing of the donor’s cells and the use of a panel of HLA-DR-matched and -mismatched antigen-presenting cells, 216-8E recognizes the peptide only in the context of HLA-DR*15:02 (Fig. S2B). Functional characterization of 216-8E was performed using intracellular staining after exposure to autologous monocyte-derived dendritic cells (moDCs) loaded with whole pneumolysoid or heat-inactivated unencapsulated TIGR4 (TIGR4_ΔCPS_). The 216-8E T-cell clone produced TNF-α, IFN-γ, and IL-17A within the first 6 h in response to whole protein and heat-inactivated TIGR4_ΔCPS_ in three independent experiments (Fig. 3B and C). These data indicate a Th1/Th17 phenotype for this predicted Ply_235–252_ epitope-specific T-cell clone.

## DISCUSSION

High-throughput arrays have been used to screen for antibodies induced against pneumococcal proteins and have identified immunogenic proteins (35, 36, 38). In contrast, basic antigen tools for CD4^+^ T-cell immunogenicity screening are more complex due to dependence on MHC class II processing and presentation and limitations in human PBMC samples and were therefore lacking to date. We evaluated a bioinformatics screening tool, which has previously successfully been used to predict HLA-DRB1-restricted immunogenic consensus sequence pathogen-derived proteins for, e.g., Helicobacter pylori and hepatitis C virus (45, 46), to identify potentially T-cell immunogenic pneumococcal protein regions. Binding motifs for eight common HLA-DRB1 alleles were applied on primary sequences of 100 pneumococcal proteins of diverse subcellular localization. The cell-mediated arm of the immune system can recognize fragments of pathogen-derived proteins of any subcellular localization, as long as they are processed and presented in the context of MHC molecules on APC. Among the top 12 T-cell immunogenic proteins and 8 (pre)clinical vaccine candidates selected for further evaluation of T-cell recognition, 14 are known cell surface proteins, 2 are secreted proteins, 3 are intracellular, and 1 protein is of unknown subcellular localization.

Using synthetic peptides for *in vitro* stimulations, we evaluated the 2 to 5 most immunogenic regions of the top 12 immunogenic proteins and 8 known vaccine candidates in PBMCs of 21 healthy adult donors. These donors were likely previously exposed to pneumococcus by a single or multiple carriage episodes throughout life. Eight of the 20 protein peptide pools elicited proliferation in over 20% of the donors. Notably, five out of these eight proteins were (pre)clinical vaccine candidates. The vaccine candidates did not have a top EpiMatrix protein score; nonetheless, these proteins showed their T-cell immunogenic potential in this study, in addition to their already-known immunogenicity, albeit mainly based on humoral responses (35, 43, 47, 50). Only the peptide pool from the amino acid ABC transporter (SP_1241) was unable to induce proliferation in any of the donors despite its predicted immunogenicity. The lack of responses to peptides of this protein (or of any other protein) could be due to low protein expression by circulating strains, poor or no processing, presentation of the epitopes by APC, or lack of T-cell repertoire.

We characterized the cell proliferation and cytokine production at the level of single peptides within the immunogenic peptide pools of BlpZ (SP_0546), PspA (SP_0117), and Ply (SP_1923). PspA and Ply, but not BlpZ, have previously been described as immunogenic targets for CD4^+^ T cells (39, 51). We now show the immunogenicity of specific epitopes within these proteins. Interestingly, among the top 5 tested immunogenic regions of PspA, two immunogenic epitopes, PspA_593–610_ and PspA_673–690_, are within the C-terminal choline-binding module of PspA and contained the typical choline-binding repeat (CBR) consensus motifs, which are highly similar between different PspA clades and other pneumococcal choline-binding proteins (CBP) (52). Indeed, all repeats of PspA were predicted to be potential CD4^+^ T-cell epitopes, and similar CBR were predicted as immunogenic epitopes in SP_2136 (PcpA) but not among PcpA’s top 5 most immunogenic regions (data not shown). The similarity of CBR within different CBP could explain the responsiveness of all three donors tested for this specific peptide (Fig. 2B). The prevalence of hydrophobic and aromatic residues in CBR likely explains the presence of multiple HLA-DR binding motifs, which frequently prefer hydrophobic and aromatic residues at anchor sites. Whether the high prevalence of the CBR consensus motif in many CBP family members underlies T-cell cross-reactivity remains to be elucidated. The immunogenic PspA_185–204_ epitope identified in our study largely overlaps a previously predicted epitope, PspA_180–199_, which was suggested to be associated with protective IL-17A responses in mice (43). In our study, PspA_185–204_ was associated with a polyfunctional cytokine response, including IL-17A in PBMCs from a single human donor. Screening of additional donors is required to further assess the incidence and functionality of human responses to this epitope.

BlpZ was not previously associated with immunogenicity, as antibodies have not been detected against this protein yet (35, 36, 38). Here, we show the strong CD4^+^ T-cell immunogenic potential of this protein, with induction of IFN-γ, IL-17A, and IL-13 for different peptides. BlpZ is a bacterial immunity protein involved in the protection against bacteriocins, which are associated with virulent pneumococcus dispersed in biofilm (48). BlpZ-specific T-cell activation in response to pneumococcal dispersion might play an important role in clearance of pneumococcus from the nasopharynx. Domain 4 of Ply has been shown to induce Th17 responses in humans (39). Here, we predicted and evaluated an immunogenic region, Ply_405–425_, within this domain, and the peptide Ply_408–425_ in this region was shown to induce the production of IL-17A after exposure to PBMCs. In addition, we predicted and evaluated immunogenic peptides outside domain 4 of Ply. In particular, Ply_236–259_ was found to induce strong proliferation in one donor, and its immunogenicity was confirmed through isolation of a specific IL-17A-producing CD4^+^ T-cell clone.

We established that reverse immunology could predict *in vivo* processed and presented immunodominant epitopes by the isolation of the HLA-DR-restricted CD4^+^ T-cell clone from an HLA-DRB1*04:03- and HLA-DRB1*15:02-typed donor, specific for the above predicted Ply epitope. Interestingly, the T-cell clone appeared restricted by HLA-DRB1*15:02 and was not able to recognize the peptide in the context of the closely related HLA-DRB1*15:01 molecules, although this allele is predicted to bind the Ply epitope. Therefore, most likely our findings indicate that the polymorphism at position β86, lining the α-helix of the peptide-binding cleft, is relevant for recognition by the T-cell receptor of clone 216-8E but less likely so for differential binding of the epitope in HLA-DRB1*15:01 and HLA-DRB1*15:02. Functionally the CD4^+^ T-cell clone was characterized as having a mixed Th1 and Th17 phenotype by the production of IFN-γ, TNF-α, and IL-17A in response to whole protein. The potential immunodominance of this particular T cell within this donor was illustrated by the stimulation of the whole PBMC fraction of this donor, which showed strongest proliferation to this particular peptide (Fig. 2D), which also induced production of IFN-γ, TNF-α, and IL-17A, in addition to the more limited production of IL-13 (Fig. 2E). Ply is present in most pneumococcal clinical isolates and promotes mucosal inflammation, increasing bacterial spread and transmission (53, 54). APC can pick up Ply and activate CD4^+^ T-cell-mediated IL-17A production. The production of IL-17A in response to pneumococcal proteins has been implied to protect against carriage through the recruitment of neutrophils that phagocytose the bacterium. However, whether this particular CD4^+^ T-cell specificity could have a role *in vivo* against carriage in humans cannot be concluded from our data. *In vivo* studies are required to show local IL-17A production via activation of epitope-specific Th17 cells in response to Ply.

Previous work by Li et al. ranked the capacity of individual pneumococcal proteins to specifically elicit Th17 T-cell responses when presented in various protein pools (44). A small number of proteins (*n* = 8) with intermediate to high antigenicity scores in the Li et al. paper were also evaluated for potential T-cell immunogenicity (EpiMatrix protein score) in our study, six of which were further validated using peptide pools in proliferation assays. This small intersection of proteins and the divergent methodologies preclude a meaningful comparison of the ranking outcome between the two studies. Also, in principle, a protein could well elicit a strong Th17 type immune response based on one particular immunodominant epitope binding to a number of HLA class II alleles but could have a low EpiMatrix protein score if it lacks further sequences with HLA class II binding motifs. Nevertheless, the lymphoproliferative responsiveness found against all six intersecting and validated proteins in our study (SP_0641, SP_1072, SP_1434, SP_1839, SP_2136, and SP_2216; Table 3) confirmed that both approaches to identify T-cell immunogenic pneumococcal proteins yield common targets.

The EpiMatrix immunogenicity score of a protein is based on all potential epitopes binding the HLA-DR alleles included in the predictions. A high EpiMatrix score suggests a high probability of T-cell responses, but a low score does not exclude that a protein could evoke T-cell responses. As mentioned, among all potential epitopes a single peptide can be immunodominant. Therefore, we characterized the cell proliferation and cytokine production at the level of single peptides within the immunogenic peptide pools of BlpZ, PspA, and Ply, having divergent overall EpiMatrix protein scores. The breadth of the responses in donors differed from a single Ply peptide in MB209 to up to five PspA epitopes in MB222. Interestingly, a single-peptide specificity could also be associated with diverse Th type responses, as shown by the production of Th1, Th2, and Th17 signature cytokines after stimulation, even in a single donor. Moreover, different donors could respond similarly or differently against single-peptide specificities. Various pathogen and host factors may drive the primary selection and functional differentiation of CD4^+^ T-cell responses against a single epitope. These include pathogen virulence, duration of infection, and immunomodulatory properties of antigens involved, in combination with a donor’s innate immune response, HLA background, and T-cell receptor repertoire (55). Further studies, including testing of more donors and response kinetics, are needed to determine if patterns exist in pneumococcal peptide- or protein-specific cytokines. The type of Th responses that can be induced to a protein/epitope may be crucial for its effectiveness *in vivo* and, hence, an important factor when developing targeted vaccine approaches.

A large diversity of HLA types, which all have various peptide binding motifs (56), is expressed by humans. The *in silico* prediction method used covered a wide range of HLA-DRB1 in four-digit typed alleles (e.g., HLA-DRB1*01:01) but did not include the other HLA class II molecules, HLA-DRB3-, -4-, or -5-encoded HLA-DR molecules, HLA-DQ, and HLA-DP, which are also capable of presenting peptides. Through this limitation, the potential HLA class II-restricted epitope of the pneumococcal protein panel may have been underestimated. On the other hand, promiscuous HLA binding potential is a feature of many class II-restricted T-cell epitopes, and putative epitopes for HLA class II often tend to cluster in particular protein regions (57). In fact, our finding that the HLA-DRB1*15:01-predicted Ply_235–252_ epitope was presented and recognized by a CD4^+^ T-cell clone in the context of HLA-DRB1*15:02 underscores such promiscuity. As another limitation, we only included the top 5 immunogenic regions of the 20 selected proteins and may have missed T-cell epitopes that scored lower in this prediction model.

In conclusion, we investigated the T-cell immunogenic potential of previously studied and unstudied pneumococcal proteins, predicted T-cell immunogenic regions by *in silico* tools, and confirmed these predictions *in vitro*. Distinct Th-type responses were induced after single-peptide PBMC stimulation and T-cell clone activation. Reverse immunology, applying *in silico* predictions together with *in vitro* testing, proved a powerful semi-high-throughput approach to identify a series of immunogenic proteins and protein regions, useful to advance the development of immunomonitoring assays and targeted vaccine approaches.

## MATERIALS AND METHODS

### Human blood and PBMC isolation.

Buffy coats were obtained from HLA class II (two-digit) typed healthy adult blood donors (Sanquin Blood Supply, Amsterdam, The Netherlands). All donors provided written informed consent in accordance with the local protocol for blood donations not for transfusion. The study was approved by the Medical Ethics Committee of Sanquin Blood Supply (Amsterdam, The Netherlands). PBMCs were isolated from buffy coats by Lymphoprep (Axis-Shield, Oslo, Norway) density gradient centrifugation. PBMCs were frozen in fetal calf serum (FCS; Greiner Bio-One, Kremsmünster, Austria) containing 10% dimethyl sulfoxide (DMSO; Sigma-Aldrich, Saint Louis, MO, USA) and stored at −135°C until use.

### *In silico* T-cell immunogenicity predictions.

*In silico* immunogenicity predictions of whole pneumococcal proteins were performed using the EpiMatrix system (EpiVax, Providence, RI) (58). Protein sequences were from TIGR4 and R6 (Table 1). Sequences of 9-mer frames, with 8 overlapping amino acids, of the proteins were evaluated for binding motifs for a panel of eight common HLA-DRB1 alleles (DRB1*01:01, DRB1*03:01, DRB1*04:01, DRB1*07:01, DRB1*08:01, DRB1*11:01, DRB1*13:01, and DRB1*15:01), considered to represent additional family members and to cover over 98% of the human population (59). Each frame for each allele is scored (−3 to +3). EpiMatrix assessment scores (Z) above 1.64 indicate a significant chance of HLA-DR binding. The EpiMatrix protein score is the difference between the number of epitopes predicted and the number of T-cell epitopes expected to be found by chance in a protein of the same size. Proteins scoring above 20 were considered to have a significant overall immunogenic potential. Potential cross-reactivity of frames against the human genome was assessed using the JanusMatrix tool, which returns a score for a given peptide cluster or protein indicating the coverage within the human genome; Janus protein scores above 3.0 and peptide regions with an aggregated Janus cluster score of >2 were considered potentially cross-reactive and, thus, nonimmunogenic (57). EpiBars, defined as 9-mer frames predicted to bind at least 4 of the 8 common HLA-DRB1 alleles, were further specified *in silico* for selected proteins. Protein regions of 15 to 25 amino acids with a high density of EpiMatrix assessment scores of >1.64 were assigned an aggregated EpiMatrix cluster score. Cluster scores of >10 were considered significant (Fig. 1 and Table 2). For selected proteins, a maximum of 5 immunogenic regions with the highest EpiMatrix cluster scores were selected for peptide synthesis based on optimal EpiBar coverage. An overview of the reverse immunology process is depicted in Fig. S1 in the supplemental material.

### Generation of synthetic peptides and protein and whole pneumococcal cell preparation.

Peptides with a maximum length of 18 amino acids were chemically synthesized. For EpiBar-based immunogenic regions longer than 18 amino acids, two partially overlapping peptides were designed (Pepscan, Lelystad, The Netherlands). In total, 160 synthetic peptides were synthesized, representing 99 potential immunogenic regions. Peptides were dissolved in DMSO at a stock concentration of 1 mM per peptide. Pools for each protein were assembled, varying from 4 to 12 peptides per pool. PlyD1, a genetically detoxified pneumolysin (T65C, G293C, and C428A), was kindly provided by M. Ochs (Sanofi-Pasteur, Swiftwater, PA, USA). TIGR4_ΔCPS_ was kindly provided by M. de Jonge (Radboud University Medical Center, Nijmegen, The Netherlands) (60) and was cultured up to an optical density of 0.6. An inactivated whole-cell preparation was prepared by 1 h of heat inactivation of the biomass at 56°C.

### HLA typing and cell lines.

Four-digit molecular typing for HLA class II alleles of blood donors was performed based on the sequence-specific oligonucleotide PCR technique in combination with Luminex using commercial reagents on PBMC-derived DNA at the Laboratory of Translational Immunology, University Medical Center Utrecht, Utrecht, the Netherlands (H. Otten and E. Spiering). Four-digit HLA-typed Epstein-Barr virus-transformed B-cell lines (B-LCL) for MHC restriction analysis were kindly provided by F. Claas (Leiden University Medical Centre).

### Immunogenicity screening of predicted peptides.

PBMCs from donors, selected to express at least 1 of the 8 common HLA-DRB1 alleles in the EpiMatrix system (based on their two-digit HLA-DRB1 typing), were stimulated in AIM-V (Gibco, Thermo Fisher Scientific, Waltham, MA, USA), supplemented with 2% human AB serum (Sigma-Aldrich) (referred to as complete AIM-V), in 96-well U-bottom plates at a concentration of 1.5 × 10^5^ cells/well in the presence of individual or pooled peptides (at 1 μM per peptide) or medium as a negative control. Stimulations were performed in quadruplicate wells and incubated for 5 days at 37°C with 5% CO_2_. Here, supernatant was harvested, pooled for quadruplicate stimulations, and stored at −20°C, and cell proliferation was determined.

### Cell proliferation.

Cell proliferation was determined after 5 days of *in vitro* stimulation by adding tritium thymidine (18 kBq/well) to the 96-well plates for overnight incubation at 37°C with 5% CO_2_ to be incorporated in the cellular DNA with every cell division. Cells were then harvested on a filter, and incorporated label was determined as counts per minute (cpm) using a MicroBeta counter (Perkin Elmer). Stimulation indices (SIs) were calculated by dividing the mean cpm of the quadruplicate stimulated wells by the mean cpm of the quadruplicate medium control wells. SIs of >1.7 were considered positive, and results are shown as mean SIs per group with standard deviations.

### Cytokine responses.

Concentrations of IL-2, IL-4, IL-5, IL-10, IL-13, IL-17, TNF-α, and IFN-γ were measured in pooled cell culture supernatants using a human cytokine kit (Merck-Millipore, Burlington, MA, USA) and multiplex technology according to instructions of the manufacturer. Samples were measured and data were analyzed with Bio-Plex200 and Bio-Plex Manager 5.0 software (Bio-Rad Laboratories).

### Cloning of Ply-specific CD4^+^ T cells.

PBMCs of donor responding to the Ply peptide pool were stimulated with PlyD1 at 1 μg/ml in complete AIM-V at 37°C in 5% CO_2_. After 7 days of expansion, stimulated T cells were diluted to nearly single cells and cultured in the presence of gamma-irradiated feeder cells at 1.5 × 10^5^ PBMC/well and phytohemagglutinin at 1 μg/ml in 96-well plates at 37°C in 5% CO_2_ for 2 to 3 weeks. Expanding T-cell cultures from plates with <36% cell outgrowth were considered potentially clonal and were evaluated for reactivity to whole protein stimulation with autologous APC before assessing the epitope specificity through peptide stimulations.

### Restriction analysis of CD4^+^ T-cell clone.

MHC restriction of Ply_235–252_-specific CD4^+^ T-cell clone 216-8E was assessed by measuring proliferation after whole protein stimulation using autologous or HLA-typed B-LCL as APCs. APCs were mock pulsed or pulsed with PlyD1 at 1 μg/ml overnight at 37°C in 5% CO_2_, washed, fixed using 0.25% paraformaldehyde solution for 10 min at room temperature, and washed with 0.2 M glycine solution. Anti-HLA-DR (B8.11-2; in-house), anti-HLA-DQ (SPV-L3; in-house), or anti-HLA-DP (B7/21; Leinco Technologies, Fenton, MO, USA) blocking antibody was used to confirm the restricting element of clone 216-8E. T-cell receptor Vα and Vβ sequencing confirmed clonality of Ply_235–252_-specific CD4^+^ T-cell clone 216-8E.

### Generation of moDCs.

PBMCs were thawed, plated at 40 × 10^6^ cells per T75 flask in Iscove’s modified defined medium (IMDM; Gibco, Life Technologies) containing 1% FCS and 1% penicillin-streptomycin (Gibco, Life Technologies), and incubated at 37°C in 5% CO_2_. Monocytes were isolated using plastic adherence and cultured as follows. Nonadherent cells were removed after 2 h, and adherent cells were washed once with phosphate-buffered saline. IMDM containing 1% FCS, 1% penicillin-streptomycin, IL-4 (500 U/ml; PeproTech, Rocky Hill, NJ, USA), and granulocyte-macrophage colony-stimulating factor (500 U/ml; PeproTech) was added to adherent cells. Monocytes were differentiated into monocyte-derived dendritic cells (moDCs) for 5 days.

### Flow cytometry.

Flow cytometric analysis was used to determine intracellular cytokines produced by the CD4^+^ T-cell clone 216-8E after 6 or 26 h of exposure to moDCs loaded with heat-inactivated TIGR4_ΔCPS_ (10^7^ culture-forming units/ml), PlyD1 (1 μg/ml), or no antigen as a control. In the last 4 h of stimulation, brefeldin A (BD Bioscience, San Jose, CA, USA) was added to capture intracellular cytokines. Cells were stained using fixable live/dead dye (ZombieNIR; BioLegend, San Diego, CA, USA) for 10 min at room temperature. After washing, surface proteins were stained using anti-human CD3 (SK7), CD4 (OKT4), and CD8 (SK1; all from BioLegend) antibodies for 20 min at 4°C. Cells were fixed and permeabilized using a fixation/permeabilization kit (eBioscience, San Diego, CA, USA) according to the manufacturer’s protocol, followed by staining with anti-human IFN-γ (B27), TNF-α (Mab11), IL-17A (BL168) (all from BioLegend), and IL-4 (MP4-25D2; BD, Franklin Lakes, NJ, USA). Cells were measured using the fluorescence-activated cell sorter LSRFortessa X-20 (BD).

### Data availability.

The data generated or analyzed in this study are available from the corresponding author on reasonable request.

## Supplementary Material

Supplemental file 1

Supplemental file 2
