# Supplementary material for: Prediction and Validation of Immunogenic Domains of Pneumococcal Proteins Recognized by Human CD4+ T Cells
Source: Infect Immun. 2019 May 21;87(6):e00098-19. doi: 10.1128/IAI.00098-19 (PMC6529658; doi:10.1128/IAI.00098-19)
Supplement: Supplemental file 1 [file IAI.00098-19-s0001.pdf]

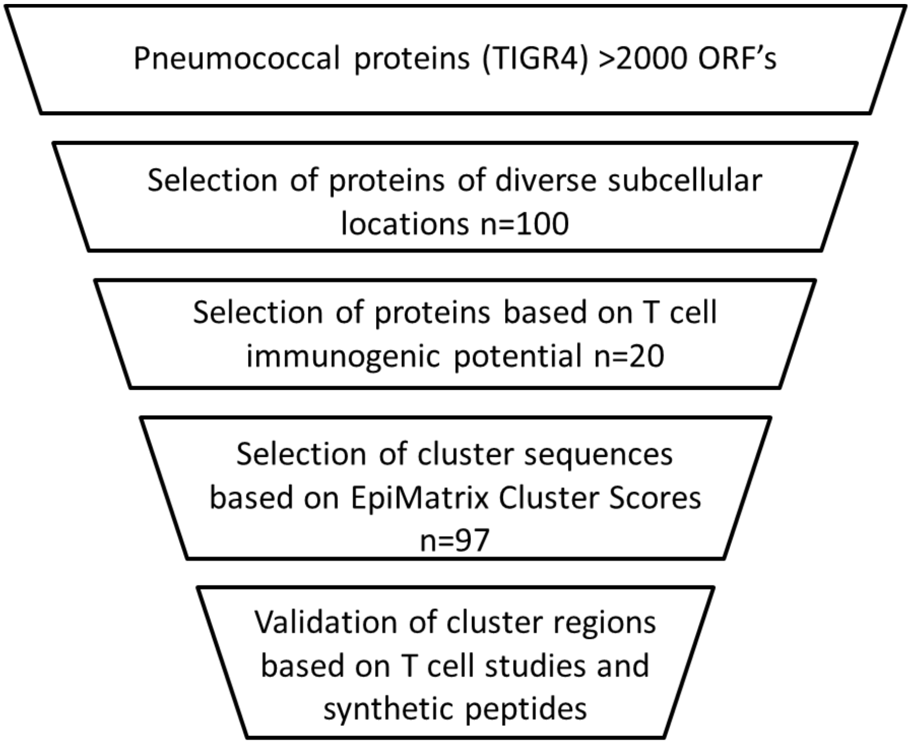

Figure S1: Overview of the reverse immunology process. Based on literature a selection of 100 pneumococcal proteins was made. Protein immunogenicity was predicted *in silico* using the EpiMatrix System. The top 12 most immunogenic proteins, together with 8 (pre)-clinical vaccine candidates, were selected for in-depth analysis. Multiple immunogenic regions in each of these 20 proteins were predicted using *in silico* tools. Up to 5 of the most immunogenic regions per protein were synthesized in maximum 18-mer peptides, resulting in a total production of 160 peptides for *in vitro* assessment.
