# Supplementary material for: Prediction and Validation of Immunogenic Domains of Pneumococcal Proteins Recognized by Human CD4+ T Cells
Source: Infect Immun. 2019 May 21;87(6):e00098-19. doi: 10.1128/IAI.00098-19 (PMC6529658; doi:10.1128/IAI.00098-19)
Supplement: Supplemental file 2 [file IAI.00098-19-s0002.pdf]

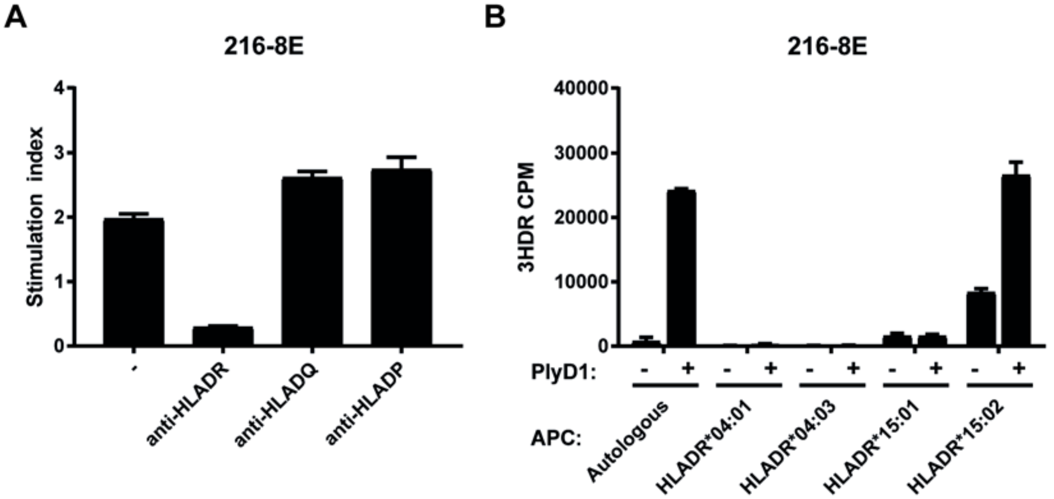

Figure S2: CD4$^+$ T cell clone 216-8E epitope recognition is restricted to HLA-DRB1*15:02. Determination of the HLA-Class II restriction of the CD4$^+$ T cell clone 216-8E epitope was performed by measuring proliferation after exposure to autologous B-LCL pulsed with PlyD1 in the presence of antibodies blocking HLA-DR, HLA-DQ or HLA-DP (A). Detailed restriction analysis was accomplished by measuring proliferation after exposure to four-digit HLA typed B-LCL as APCs pulsed with PlyD1 (B).
